# The Appropriate Use of Testing for COVID-19

**DOI:** 10.5811/westjem.2020.4.47370

**Published:** 2020-04-13

**Authors:** Tony Zitek

**Affiliations:** Kendall Regional Medical Center, Department of Emergency Medicine, Miami, Florida; Nova Southeastern University, Dr. Kiran C. Patel College of Allopathic Medicine, Davie, Florida

## Abstract

Many public officials are calling for increased testing for the 2019 novel coronavirus disease (COVID-19), and some governments have taken extraordinary measures to increase the availability of testing. However, little has been published about the sensitivity and specificity of the reverse transcriptase-polymerase chain reaction (RT-PCR) nasopharyngeal swabs that are commonly used for testing. This narrative review evaluates the literature regarding the accuracy of these tests, and makes recommendations based on this literature. In brief, a negative RT-PCR nasopharyngeal swab test is insufficient to rule out COVID-19. Thus, over-reliance on the results of the test may be dangerous, and the push for widespread testing may be overstated.

## INTRODUCTION

A novel coronavirus disease (COVID-19), caused by SARS-CoV-2, has rapidly spread throughout many countries including the United States since its discovery in December 2019.^1^ Many locations in the US are looking to rapidly expand their testing capabilities for this virus as they believe this could provide an important means to battle the COVID-19 pandemic.^2^–^6^ However, the benefit of widespread testing depends on the accuracy of the test, and how the results of the test will affect treatment. For mild cases of COVID-19 (which are the primary target of the outpatient testing facilities), no specific medications are indicated, so in most cases, the results of the test would not change treatment. With regard to the accuracy of the test, the most commonly used test for detecting SARS-CoV-2 is a nasopharyngeal swab that uses a reverse transcriptase-polymerase chain reaction (RT-PCR) to identify viral RNA. Data from in vitro analyses suggest that the RT-PCR test is highly specific for SARS-CoV-2, as it is not positive when exposed to the nucleic acid of other common viruses.^7^ Similarly, the in vitro sensitivity of RT-PCR tests is high, but in clinical settings the sensitivity of the nasopharyngeal RT-PCR swab tests for diagnosing COVID-19 is questionable. This article will review the clinical data regarding the accuracy of the COVID-19 RT-PCR test.

## SUMMARY OF THE LITERATURE REGARDING COVID-19 TESTING

At this time, no peer-reviewed publications have reported a sensitivity and specificity of RT-PCR tests for COVID-19. One non-peer reviewed publication reports that, based on 87 Chinese patients who were ultimately diagnosed with COVID-19, pharyngeal RT-PCR tests have a sensitivity and specificity of 78.2% and 98.8%, respectively.^8^ The sensitivity was 62.5% for “mild” cases.^8^ While no other publications currently provide estimates of the sensitivity and specificity, several peer-reviewed publications have provided evidence of a substantial false negative rate with RT-PCR swab tests as described below.

First, a study by Wang et al took various types of specimens from 205 patients with confirmed COVID-19 and tested them with RT-PCR. Of 398 pharyngeal swabs, they found only 126 (32%) were positive. They took just eight nasal swabs, and found five (63%) were positive.^9^ (As a side note, the US Centers for Disease Control and Prevention has reported that nasopharyngeal swabs seem to be more sensitive than oropharyngeal swabs, and thus recommends nasopharyngeal testing over oropharyngeal testing.^10^) Wang et al also analyzed specimens from bronchoalveolar lavage (BAL) fluid and sputum and found these were positive in 93% and 72% of cases, respectively.^9^

Along the same lines, Winichakoon et al published a letter to the editor in which they described a case of a COVID-19 patient who had a nasopharygeal/oropharyngeal RT-PCR swab that was negative for COVID-19, but RT-PCR of BAL fluid was positive.^11^ Additionally, 19 cases of patients with suspected COVID-19 were reviewed in another small study. Oropharyngeal RT-PCR swab tests were performed in all 19 patients, but were positive in just nine (47.4%).^12^

Next, in a case series described by Xie et al, five patients from the Hunan province of China had ground-glass opacities on chest computed tomography (CT) that were suggestive of COVID-19, but initial pharyngeal RT-PCR tests were negative. Repeat RT-PCR swabs ended up being positive.^13^ Similarly, Fang et al analyzed 51 patients who were ultimately confirmed to have COVID-19 who had both a chest CT and RT-PCR testing by either throat swab (45 patients) or sputum (six patients) upon admission to the hospital. Of those 51 patients, the chest CTs had characteristic findings of COVID-19 in 50 (98%). Comparatively, the initial RT-PCR test was positive in 36 of 51 (71%).^14^

Other studies have also demonstrated that initial RT-PCR tests may be negative and then become positive with repeated tests. For example, Wu et al studied the clinical course of 80 patients from the Jiangsu Province who were ultimately diagnosed with COVID-19. Nine of those 80 patients (11.3%) had two negative RT-PCR nasal or oral swabs before their third swabs came back positive.^15^ Additionally, Young et al reported the results from daily nasopharyngeal RT-PCR testing that were taken from 18 patients from Singapore who were hospitalized for COVID-19. Interestingly, some patients had positive tests, and then negative tests, and then positive tests again, all within the same hospitalization.^16^

## DISCUSSION

The sensitivity and specificity of nasopharyngeal swabs using RT-PCR for the diagnosis of COVID-19 cannot be precisely determined with the published data to this point. However, the available in vitro data along with minimal clinical data suggest that the test has very high specificity. On the other hand, the sensitivity is moderate (perhaps between 63–78%). Among the various ways of performing RT-PCR, pharyngeal swabs seem to have lowest sensitivity; nasal swabs may be a bit more sensitive than pharyngeal swabs. RT-PCR analysis of BAL fluid seems to be the most accurate means of virologic confirmation, but BAL fluid can only reasonably be collected on the sickest cohort of patients. For patients with moderate to severe COVID-19 symptoms, identifying characteristic findings on CT imaging of the chest may be more sensitive than RT-PCR testing.

Given these findings, when a patient has a high pretest probability for COVID-19, a negative test does not rule out the disease. Consequently, policies that assume a high accuracy of RT-PCR testing are perilous. For example, employers should not use a negative test result to decide when someone should return to work. Meanwhile, the perceived need for increased testing propagated by the popular media^17^ may lead some patients to visit the ED solely for an unnecessary test, which could put those individuals at increased risk for COVID-19 if they do not already have it. As there is no treatment needed for mild cases of COVID-19, patients with mild symptoms need not go to the emergency department or get testing; instead, they should self-quarantine.

Increased testing could be beneficial in areas of the world where there are very few cases of COVID-19. Aggressive early testing could allow for early identification of cases to allow for early targeted isolation and social distancing measures. However, in cities where COVID-19 is already widespread, the testing of large numbers of individuals with mild illness will have minimal effect on treatment but will require massive resources. There is epidemiological benefit to testing, but in cities already being devastated by COVID-19, the numbers of hospitalizations and mortalities associated with it can be used as indicators of disease impact. Reduced testing of patients with mild disease could save testing materials so that sicker patients and healthcare professionals will have access to testing. Additionally, a large amount of personal protective equipment could be saved by not attempting to test the many thousands and perhaps what will be millions of mild COVID-19 cases.

## CONCLUSION

While the exact sensitivity and specificity of RT-PCR tests for COVID-19 are not known, it appears that a positive test is highly suggestive of true COVID-19, but a negative test does not rule out the disease. Patients and providers in epidemic areas should assume they have the disease if they have the signs and symptoms of the disease even if their test was negative. The push for increased testing in areas that already have widespread COVID-19 may be overstated, as the benefits of large-scale use of a moderate sensitivity test are minimal.
